# Repeated measures of coffee consumption and risk of future incident venous thromboembolism—the Trøndelag Health Study and the Tromsø study

**DOI:** 10.1016/j.rpth.2025.103019

**Published:** 2025-08-19

**Authors:** Melliane Muteba Olsen, Sigrid K. Brækkan, Kristian Hveem, Kjersti Grønning, John-Bjarne Hansen

**Affiliations:** 1 Nord-Trøndelag Hospital Trust, Norway; 2 Thrombosis Research Group (TREC), Department of Clinical Medicine, UiT – The Arctic University of Norway, Tromsø, Norway; 3 Thrombosis Research Center (TREC), Division of Internal Medicine, University Hospital of North Norway, Tromsø, Norway; 4 HUNT Center for Molecular and Clinical Epidemiology, Department of Public Health and Nursing, Faculty of Medicine and Heath Science, Norwegian University of Science and Technology, Trondheim, Norway; 5 Department of Research, St. Olav’s Hospital, Trondheim, Norway; 6 Department of Public Health and Nursing, Norwegian University of Science and Technology, Trondheim, Norway

**Keywords:** coffee, deep vein thrombosis, pulmonary embolism, risk factor, venous thromboembolism

## Abstract

**Background:**

A limited number of epidemiological studies have reported mixed results on the association between coffee consumption and risk of venous thromboembolism (VTE).

**Objectives:**

We aimed to investigate the association between repeated measures of coffee consumption over time and risk of incident VTE in a large population-based cohort.

**Methods:**

Participants (*N* = 112,784) were recruited from 4 surveys of the Tromsø Study (enrolment: 1994-2008) and 2 surveys of the Trøndelag Health Study (enrolment: 1995-2008) and followed through 2020. Information on coffee consumption and major confounders (age, sex, body mass index, physical activity, arterial cardiovascular diseases, and cancer) was updated at each survey. Time-varying Cox regression models were used to calculate hazard ratios (HRs) for VTE across categories of coffee consumption.

**Results:**

There were 178,696 observation periods and 3419 VTEs during follow-up. A threshold effect was observed, and those who drank 1 to 2 cups of coffee per day had 21% lower risk of overall VTE (HR, 0.79; 95% CI, 0.68-0.93) than nonconsumers. The inverse association of coffee consumption (1-2 cups/d) with VTE was more pronounced for pulmonary embolism (HR, 0.72; 95% CI, 0.58-0.89) than for deep vein thrombosis (HR, 0.87; 95% CI, 0.70-1.09). The HRs for VTE remained similar across categories of higher coffee consumption after adjustments for major potential confounders. The association of coffee consumption with VTE risk was similar in women and men.

**Conclusion:**

Coffee consumption was associated with a nonlinear lower risk of overall VTE and, in particular, pulmonary embolism.

## Essentials

• Studies on coffee consumption and risk of VTE have shown mixed results.
• We investigated the association between coffee consumption and risk of VTE in a cohort of 112,784 individuals.
• Those who drank 1 to 2 cups of coffee per day had 21% lower risk of VTE than nonconsumers.
• Coffee consumption was associated with a lower risk of overall VTE and, in particular, PE.

## 1 Introduction

Venous thromboembolism (VTE) is a common term for deep vein thrombosis (DVT) and pulmonary embolism (PE). VTE is a highly prevalent disease that annually affects almost 10 million individuals worldwide [1]. VTE is associated with severe short- and long-term complications that can seriously impair mobility and quality of life, including VTE recurrence, postthrombotic syndrome, post-PE syndrome, chronic pulmonary hypertension, and death [[2], [3], [4], [5]]. In contrast to arterial cardiovascular diseases (CVDs), such as myocardial infarction and stroke, where the incidence has declined during the last decades [6,7], time trend studies have shown a slight increase in the incidence of VTE during the same period [8,9]. It is assumed that the incidence of VTE will continue to rise since major VTE risk factors, such as obesity, cancer, and the proportion of elderly, are all increasing in Western societies [10,11]. Therefore, it is imperative to identify individual lifestyle factors that protect against VTE to promote lifestyle changes that potentially can reduce the burden of VTE in society.

Coffee is one of the most widely consumed alcohol-free beverages in the world [12,13], and Norway is one of the countries with the highest coffee consumption rates, with an average of >3 cups of coffee daily per adult [14]. Coffee intake has been linked to certain health concerns, such as increased blood pressure [15], higher plasma homocysteine [16,17], high lipoprotein concentration in the blood [18], increased risk of arterial CVDs [19,20], and deterioration of sleep quality [21]. However, cohort studies have shown a 12% lower risk of all-cause mortality in the general population for those in the highest vs the lowest category of coffee consumption [22]. Meta-analyses also reported that the intake of at least 4 cups of coffee per day might have a protective effect on the incidence and mortality of many chronic diseases [[23], [24], [25]].

Two previous cohort studies have reported an inverse and U-shaped association between coffee consumption and risk of future incident VTE, where moderate consumption (4-7 cups per day) of coffee was associated with a 12% to 33% lower risk of VTE [26,27]. These findings were confirmed in a large case-control study, the Multiple Environmental and Genetic Assessment of risk factors for venous thrombosis (MEGA) study [28]. Recently, however, Yuan et al. [29] found no association between coffee consumption and VTE risk in a Swedish population-based cohort. Coffee habits (eg, espresso, boiled, and filtered) and amounts vary between countries [[30], [31], [32]] and over time [33,34]. In addition, coffee consumption is associated with other lifestyle patterns, such as physical activity [35,36] and smoking [37], which may influence VTE risk [38].

The objective of this study was to investigate the association between repeated measures of coffee consumption over time and risk of VTE in a general population when accounting for other related lifestyle factors.

## 2 Methods

### 2.1 Study population

The study population included all participants from the fourth to the seventh survey of the Tromsø Study (conducted in 1994-1995, 2001-2002, 2007-2008, and 2015-2016, respectively), as well as the second and third (1995-1997 and 2006-2008, respectively) survey of the Trøndelag Health (HUNT) Study. Both the Tromsø and HUNT studies are cohorts based on the general Norwegian population, comprising residents of Tromsø Municipality and the former Nord-Trøndelag County, respectively. A total of 36,626 women and men, aged between 25 and 99 years, participated in at least 1 of the Tromsø 4 to 7 studies [39,40], while 78,959 women and men aged 19 to 100 years attended at least 1 of the HUNT surveys [41,42]. These 2 cohorts were combined into a large cohort, including a total of 115,585 individuals. We excluded participants with a known history of VTE (*n* = 223), participants who officially moved out of the study area before inclusion (*n* = 87), and participants with missing information on coffee consumption (*n* = 2491). In total, 112,784 unique individuals were included in the study, contributing to a total of 178,696 observation periods, since some of the participants attended >1 survey, and information on coffee consumption was recollected (Figure).

**Figure:**
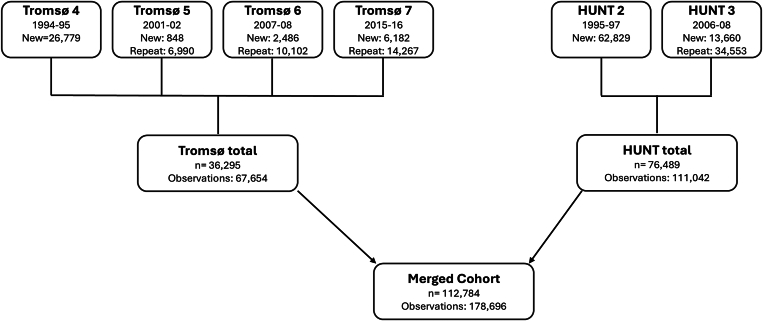
Flowchart of study participants who provided information on coffee consumption in the different Tromsø (4-7) and Trøndelag Health (HUNT2-3) study surveys. “New” indicates number of newly recruited participants who provided information on coffee consumption at the survey. “Repeat” indicates number of participants who had coffee consumption remeasured at the survey. Observations: New + Repeat.

The study was approved by the Regional Committee for Health and Research Ethics in North Norway (Approval number 424360), and all participants gave informed and written consent to participate.

### 2.2 Baseline measurements

The design and data collection were very similar between the 2 cohorts [[39], [40], [41], [42]]. Information about the participants was collected at each survey by questionnaires (self-report), physical examination, and blood samples. The physical examination included measurements of body height and weight, and body mass index (BMI) was calculated. Information on prevalent CVDs (including myocardial infarction, stroke, and angina), cancer, physical activity, smoking habits, and coffee consumption was collected through questionnaires. The questions on coffee intake were as follows: “How many cups of coffee do you usually drink daily?” and the participants were asked to fill in the number of cups of “boiled coffee” (coarsely ground coffee for brewing) or “other types of coffee” consumed per day. Based on this information, we generated a variable for total daily coffee consumption, and categorized it as follows: “no coffee consumption,” “1-2 cups per day,” “3-4 cups per day,” “5-6 cups per day,” and “> 6 cups per day.” A dichotomous physical activity variable was created, where the active group comprised participants reporting physical activity ≥ 1 hour per week, and the inactive group those reporting no physical activity or < 1 hour per week, regardless of intensity.

### 2.3 VTE identification and validation

All VTE events occurring during follow-up in participants in Tromsø and HUNT were identified by searching the registries of the relevant referral hospitals (ie, the University Hospital of North Norway in Tromsø, and the 2 local hospitals Levanger and Namsos, as well as the regional tertiary-care center, St. Olavs Hospital in Trondheim), as previously described [8,43]. The medical record of each identified VTE event was carefully reviewed by trained healthcare personnel (who were blinded to the survey information from the Tromsø and HUNT studies), and a VTE event was adjudicated and recorded when signs and symptoms of DVT or PE were followed by objective confirmation of a thrombus by radiological imaging, and anticoagulant treatment was initiated.

A VTE event was categorized as either unprovoked or provoked (ie, ≥1 provoking factor) based on the presence of provoking factors at the time of diagnosis. These provoking factors included recent surgery or trauma within the past 8 to 12 weeks, acute medical conditions (acute myocardial infarction, ischemic stroke, or a major infectious disease), active cancer, significant immobilization (defined as bed rest for >3 days or wheelchair use within 8 weeks before the event, or long-distance travel of >4 hours within 2 weeks prior to the event), or other provoking factor(s) specifically noted by a physician in the medical record (eg, an intravascular catheter).

### 2.4 Statistical analysis

Stata version 18.1 (StataCorp) was used to perform statistical analyses. Each participant was followed from the date of inclusion in the Tromsø or HUNT surveys until the occurrence of a first lifetime VTE, migration, death, or the end of the follow-up period (December 31, 2019, in HUNT and December 31, 2020, in Tromsø), whichever came first. As many participants attended several of the surveys, and thereby had repeated measurements of coffee consumption and other characteristics, we used a time-varying analysis where individuals who participated in multiple surveys contributed 1 observation period per survey attended. This resulted in a total of 178,696 observation periods (Figure).

Summary statistics were used to present characteristics of the study population (observation periods) across categories of coffee consumption. Age-adjusted incidence rates (IRs) were estimated for each coffee consumption category using Poisson regression and expressed as number of events per 1000 person-years. Cox proportional hazards regression models, with coffee consumption as a time-varying covariate, were used to estimate hazard ratios (HR) with 95% Cls for VTE after adjustment for age and sex (model 1), and age, sex, BMI, daily smoking (yes/no), history of CVDs, and cancer (model 2). The adjustment factors were also updated at each survey and entered as time-varying covariates in the models to optimize confounder adjustment. The degree of missingness of any of these covariates was <1%, and complete case analyses were performed in the multivariable models. We additionally performed analyses stratified by sex. For subgroup analyses, all regression models were repeated with PE, DVT, unprovoked VTE, and provoked VTE as outcomes, respectively. We also conducted sensitivity analysis with data restricted to those individuals with registered information on physical activity (*n* = 158,670 observation periods) to assess the impact of physical activity on the relationship between coffee consumption and VTE risk. In all Cox analyses, we treated death as a censoring event. Of note, the age-adjusted mortality rates did not differ substantially between the coffee consumption categories, indicating that the censoring was noninformative (ie, competing risk of death would not be an issue).

## 3 Results

Participant characteristics at the start of each observation period are presented in Table 1. Those who reported no coffee consumption were substantially younger than those who reported coffee consumption (41 years vs 50-56 years), and the proportion of women decreased across increasing coffee consumption categories (63% among nonconsumers vs 40% in those drinking >6 cups daily). Furthermore, the proportion of smokers increased across categories of coffee consumption, from 12% in nonconsumers to 46.5% in those who consumed most coffee per day. The mean BMI and proportion of CVDs and cancer were lowest among those with no coffee consumption. However, the proportion of individuals with CVDs and cancer decreased across categories with higher coffee consumption.

**Table 1:** Baseline characteristics of the Trøndelag Health Study (HUNT) and Tromsø Study participants across categories of coffee consumption.

Characteristics	No coffee consumption % (*n*)	1-2 cups/d % (*n*)	3-4 cups/d % (*n*)	5-6 cups/d % (*n*)	> 6 cups/d % (*n*)
**Total**					
No. of observations	16,507	27,747	54,487	46,148	33,807
Age (y), mean ± SD	41 ± 17	54 ± 18	56 ± 16	53 ± 14	50 ± 13
Women	63 (10,465)	61 (16,792)	58 (31,323)	50 (23,250)	40 (13,482)
BMI (kg m^−^^2^), mean ± SD	26.1 ± 4.8	26.5 ± 4.4	26.6 ± 4.2	26.7 ± 4.1	26.6 ± 4.2
Smoking	12.0 (1974)	9.3 (2584)	14.5 (7915)	27.5 (12,697)	46.5 (15,715)
CVD	3.7 (615)	9.2 (2546)	8.6 (4699)	7.3 (3367)	6.1 (2057)
Cancer	2.6 (433)	5.3 (1476)	5.2 (2804)	4.2 (1921)	3.4 (1145)
**Tromsø**					
No. of observations	5526	9409	20,341	18,101	14,277
Age (y), mean ± SD	45 ± 15	55 ± 16	56 ± 14	54 ± 14	51 ± 13
Women	61 (3357)	63 (5909)	59 (11,998)	51 (9147)	39 (5551)
BMI (kg m^−^^2^), mean ± SD	26.0 ± 4.9	26.2 ± 4.4	26.3 ± 4.3	26.4 ± 4.1	26.4 ± 4.2
Smoking	11.9 (656)	8.3 (780)	13.0 (2651)	24.4 (4421)	42.7 (6100)
CVD	3.4 (189)	7.7 (726)	6.8 (1379)	6.5 (1183)	5.5 (789)
Cancer	3.0 (165)	4.8 (455)	4.9 (990)	4.1 (745)	3.4 (480)
**HUNT**					
No. of observations	10,981	18,338	34,146	28,047	19,530
Age (y)	38 ± 17	53 ± 19	55 ± 16	52 ± 15	49 ± 13
Women	65 (7108)	59 (10,883)	57 (19,325)	50 (14,103)	41 (7931)
BMI (kg m^−^^2^)	26.1 ± 4.8	26.6 ± 4.3	26.8 ± 4.1	26.8 ± 4.1	26.7 ± 4.1
Smoking	12.0 (1318)	9.8 (1804)	15.4 (5264)	29.5 (8276)	49.2 (9615)
CVD	3.9 (426)	9.9 (1820)	9.7 (3320)	7.8 (2184)	6.5 (1268)
Cancer	2.4 (268)	5.6 (1021)	5.3 (1814)	4.2 (1176)	3.4 (665)

BMI, body mass index; CVD, cardiovascular disease; HUNT, Trøndelag Health Study.

The characteristics of study participants with VTE at the time of diagnosis are shown in Table 2. The mean ± SD age at VTE diagnosis was 70 ± 14 years, and 51% of the participants were women. DVT accounted for 52.8% of the events, while PE accounted for 47.2%. Almost 55% of the VTE events had ≥1 provoking factors, with active cancer, immobility, and surgery as the most frequent provoking factors. The characteristics of the VTE events were similar in the HUNT and Tromsø studies (Table 2).

**Table 2:** Characteristics of the incident venous thromboembolism events (*n* = 3419) during follow-up. The Trøndelag Health Study (HUNT) and Tromsø study, 1994 to 2020.

Characteristics	Total (*N* = 3419) % (*n*)	HUNT (*n* = 2345) % (*n*)	Tromsø (*n* = 1074) % (*n*)
Age at VTE (y), mean ± SD	70 ± 14	70 ± 14	69 ± 13
Women	51 (1734)	51 (1189)	51 (545)
DVT	52.9 (1807)	51.6 (1211)	55.5 (596)
PE	47.2 (1612)	48.4 (1134)	44.5 (478)
Unprovoked VTE	44.8 (1532)	46.2 (1083)	41.8 (449)
Provoked VTE	55.2 (1887)	53.8 (1262)	58.2 (625)
Surgery	16.7 (569)	17.5 (411)	14.7 (158)
Trauma	11.3 (385)	12.2 (286)	9.2 (99)
Active cancer	21.8 (746)	20.6 (484)	24.4 (262)
Acute medical condition	5.5 (189)	2.5 (58)	12.2 (131)
Immobility	21.3 (727)	21.3 (500)	21.1 (227)
Other	3.3 (114)	3.0 (72)	3.9 (42)

DVT, deep vein thrombosis; HUNT, Trøndelag Health Study; PE, pulmonary embolism; VTE, venous thromboembolism.

Table 3 displays the age-adjusted IRs and HRs for overall VTE, provoked VTE, and unprovoked VTE across categories of daily coffee consumption. There was a threshold effect of ≥ 1 cup of coffee per day, and those who reported 1 to 2 cups of coffee per day had a 21% lower risk of overall VTE compared with those who did not drink coffee when adjusted for age and sex (model 1). The HRs for overall VTE remained essentially similar across categories of higher coffee consumption. Further adjustment for BMI, smoking, CVDs, and cancer (model 2) had minor impact on the risk estimates. The IRs and HRs for overall VTE, provoked VTE, and unprovoked VTE across categories of daily coffee consumption were essentially similar for women (Supplementary Table 1) and men (Supplementary Table 2).

**Table 3:** Age-adjusted incidence rates and hazard ratios for venous thromboembolism by categories of daily coffee consumption. The Trøndelag Health Study (HUNT) and Tromsø study, 1994 to 2020.

Characteristics	Person-years	VTE	Age-adjusted IR (95% CI)^a^	Model 1 HR (95% CI)	Model 2 HR (95% CI)
**Total VTE**					
0 cup/d	198,289	237	2.39 (2.08-2.70)	Ref.	Ref.
1-2 cups/d	295,275	570	1.93 (1.77-2.09)	0.79 (0.68-0.93)	0.81 (0.69-0.94)
3-4 cups/d	575,036	1141	1.91 (1.80-2.02)	0.79 (0.68-0.91)	0.78 (0.67-0.90)
5-6 cups/d	508,955	910	2.07 (1.93-2.20)	0.84 (0.73-0.97)	0.81 (0.70-0.94)
> 6 cups/d	392,801	561	2.07 (1.89-2.24)	0.84 (0.72-0.97)	0.77 (0.66-0.90)
**Provoked VTE**					
0 cup/d	198,284	121	1.22 (1.00-1.44)	Ref.	Ref.
1-2 cups/d	295,267	286	0.97 (0.86-1.08)	0.77 (0.62-0.95)	0.76 (0.62-0.95)
3-4 cups/d	575,020	658	1.10 (1.02-1.19)	0.88 (0.72-1.07)	0.85 (0.70-1.04)
5-6 cups/d	508,941	521	1.19 (1.08-1.29)	0.94 (0.77-1.15)	0.88 (0.72-1.08)
> 6 cups/d	392,890	301	1.11 (0.98-1.24)	0.89 (0.72-1.10)	0.79 (0.63-0.98)
**Unprovoked VTE**					
0 cup/d	198,284	116	1.16 (0.95-1.38)	Ref.	Ref.
1-2 cups/d	295,267	284	0.96 (0.85-1.07)	0.82 (0.66-1.02)	0.85 (0.68-1.07)
3-4 cups/d	575,020	483	0.81 (0.74-0.88)	0.69 (0.56-0.85)	0.70 (0.57-0.86)
5-6 cups/d	508,941	389	0.88 (0.79-0.97)	0.74 (0.60-0.91)	0.73 (0.59-0.90)
> 6 cups/d	392,890	260	0.95 (0.84-1.07)	0.78 (0.63-0.97)	0.75 (0.59-0.94)

Model 1: adjusted for age and sex.

Model 2: adjusted for age, sex, body mass index, smoking, cardiovascular disease, and cancer.

HR, hazard ratio; IR, incidence rate; Ref., reference; VTE, venous thromboembolism.

a Age-adjusted incidence rates per 1000 person-years.

In sensitivity analysis restricted to those with information on physical activity, we observed that the proportion of inactive individuals increased with higher categories of coffee consumption (Supplementary Table 3), but additional adjustment for physical activity in adjustment model 3 did not influence the risk of overall VTE (Supplementary Table 4). The threshold effect, HRs, and impact of multivariable adjustments on provoked and unprovoked VTE across categories of coffee consumption were similar to that observed for overall VTE (Table 3 and Supplementary Table 4).

The IRs and HRs for DVT and PE across categories of coffee consumption are shown in Table 4. The risk estimates hardly changed across the adjustment models (Table 4 and Supplementary Table 3). The HRs for PE by low (1-2 cups/d; HR, 0.72; 95% CI, 0.58-0.90) or high (> 6 cups/d; HR, 0.64; 95% CI, 0.51-0.80) coffee consumption were significantly reduced compared with those of no coffee consumption in the multivariable-adjusted model. The corresponding HRs for DVT were 0.89 (95% CI, 0.71-1.11) for low coffee consumption and 0.90 (95% CI, 0.72-1.13) for high coffee consumption. The relationship between coffee consumption and DVT and PE risk displayed the same threshold pattern as for overall VTE, in which the association appeared already at low coffee consumption (1-2 cups/d) compared with no coffee consumption.

**Table 4:** Incidence rates and hazard ratios for deep vein thrombosis and pulmonary embolism by categories of daily coffee consumption. The Trøndelag Health Study (HUNT) and Tromsø study, 1994 to 2020.

Characteristics	Person-years	VTE	Age-adjusted IR (95% CI)^a^	Model 1 HR (95% CI)	Model 3 HR (95% CI)
**DVT**					
0 cup/d	198,284	115	1.13 (0.92-1.34)	Ref.	Ref.
1-2 cups/d	295,267	290	0.98 (0.87-1.10)	0.87 (0.70-1.09)	0.89 (0.71-1.11)
3-4 cups/d	575,020	601	1.01 (0.93-1.09)	0.89 (0.73-1.09)	0.88 (0.72-1.08)
5-6 cups/d	508,941	482	1.09 (0.99-1.18)	0.94 (0.77-1.16)	0.91 (0.74-1.12)
> 6 cups/d	392,890	319	1.15 (0.02-1-28)	0.98 (0.79-1.22)	0.90 (0.72-1.13)
**PE**					
0 cup/d	198,284	122	1.26 (1.03-1.49)	Ref.	Ref.
1-2 cups/d	295,267	280	0.94 (0.83-1.06)	0.72 (0.58-0.89)	0.72 (0.58-0.90)
3-4 cups/d	575,020	540	0.90 (0.83-0.98)	0.69 (0.57-0.84)	0.68 (0.56-0.84)
5-6 cups/d	508,941	428	0.98 (0.89-1.08)	0.75 (0.61-0.92)	0.72 (0.59-0.88)
> 6 cups/d	392,890	242	0.91 (0.79-1.03)	0.70 (0.56-0.87)	0.64 (0.51-0.80)

Model 1: adjusted for age and sex.

Model 2: adjusted for age, sex, body mass index, smoking, cardiovascular disease, and cancer.

DVT, deep vein thrombosis; HR, hazard ratio; IR, incidence rate; PE, pulmonary embolism; Ref., reference; VTE, venous thromboembolism.

a Age-adjusted incidence rate per 1000 person-years.

## 4 Discussion

We investigated the association between self-reported coffee consumption and risk of future overall VTE and VTE subtypes in a large population-based cohort with repeated measurements of the exposure of coffee consumption and major confounders, which, to some extent, would capture changes in coffee consumption (ie, amount of coffee), and with validated VTE events. The mean age and the proportion of individuals with CVDs and cancer were higher in those who drank 1 to 2 cups of coffee per day compared with nonconsumers. The proportions of men and smokers increased, while proportions of physical activity, CVDs, and cancer decreased across increasing categories of coffee consumption. The relationship between coffee consumption and overall VTE risk displayed a nonlinear threshold pattern, where those who drank ≥ 1 cup of coffee per day had 19% to 23% lower risk of overall VTE compared with nonconsumers in the multivariable-adjusted model. The inverse association between coffee consumption and VTE risk was similar for provoked and unprovoked events and was mainly driven by a lower risk of PE. High consumption of coffee (> 6 cups/d) was associated with a 36% lower risk of PE after multivariable adjustments. Our findings suggest that coffee consumption may potentially lower the risk of future VTE and, in particular, PE.

Few previous studies have investigated the association between coffee consumption and VTE risk. In the Iowa Women’s Health Study, 1950 VTE events were identified during 19 years of follow-up in 37,393 middle-aged women, and coffee consumption was associated with a 3% to 14% nonsignificant reduced risk of VTE [27]. In a population-based cohort from Norway [26], including 26,755 men and women aged 25 to 97 years, 462 VTE events occurred during 12.5 years of follow-up. The authors reported that moderate coffee consumption (3-6 cups/d) was associated with an approximately 30% lower VTE risk, with similar risk estimates for provoked and unprovoked events. Subsequently, the MEGA case-control study of 1803 young and middle-aged VTE patients and an equal number of partner controls reported that ≥ 1 cup of coffee per day consumption was associated with a 25% lower risk of VTE [28]. In a Swedish cohort, including 66,330 middle-aged men and women, of which 3827 were diagnosed with VTE during 17 years of follow-up, moderate coffee consumption (1-4 cups/d) was associated with a nonsignificant 11% to 12% reduced risk of VTE in men, while high coffee consumption (> 4 cups/d) was associated with a 22% elevated VTE risk in women [29]. In the present large population-based cohort of adult men and women with a wide age range, we found that the association of coffee consumption with VTE risk displayed a nonlinear threshold pattern that was essentially similar in men and women, and for provoked and unprovoked VTE events. However, the inverse association of coffee consumption with VTE risk was apparently stronger for PE than for DVT.

The somewhat mixed results from previous studies on the association between coffee consumption and VTE risk may be due to varying study designs, populations, confounders, and ascertainment of the outcome (VTE). In general, nondifferential misclassifications of either the exposure (ie, coffee consumption) or the outcome (ie, VTE) would lead to an underestimation of the true association [44]. All previous observational studies on the association between coffee consumption and VTE risk used self-reported questionnaires to categorize the amounts of coffee consumption at study inclusion. Self-reported coffee consumption has been demonstrated to have high validity [45]. However, lifestyle habits such as coffee consumption are highly modifiable. By measuring coffee consumption only at inclusion, a possible change in the amount of coffee consumption during follow-up would not be captured and could lead to nondifferential misclassification of the exposure, with subsequent mitigation of the real association [44]. In the present and previous studies with validated VTE events [26,28], moderate coffee consumption was associated with significantly lower VTE risk, while cohort studies solely using International Classification of Diseases (ICD) codes for ascertainment of VTE diagnosis showed a nonsignificantly lower risk [27,29] or slightly elevated VTE risk in middle-aged women [29]. As ICD codes for VTE diagnosis are known to be inaccurate and lead to nondifferential misclassification [46,47], it is likely that the association between coffee consumption and VTE risk is underestimated in studies using ICD codes for VTE diagnosis.

Confounding is a potential challenge in cohort studies due to their nonrandomized nature. Theoretically, personal characteristics differently distributed between coffee abstainers and coffee consumers may explain the observed inverse association between coffee consumption and risk of future VTE rather than being a true association. In the present cohort, however, coffee abstainers displayed lower age, physical inactivity, BMI, and prevalence of CVDs and cancer at study inclusion than those consuming 1 to 2 cups of coffee daily, all of which are associated with VTE risk [38]. In addition, the proportion of smokers increased, whereas the proportion of women, physically active, and the prevalence of CVDs and cancer decreased across higher categories of self-reported coffee consumption. Despite taking these potential confounders into account in our multivariable-adjusted models, our results showed a lower VTE risk in coffee consumers. However, it is impossible to exclude the option that the apparent protective effect of coffee consumption on VTE risk is explained by residual confounding (ie, unmeasured and unrecognized confounders) due to the observational nature of our study. The latter notion is supported by the results of a Mendelian randomization study [48] using data from the UK Biobank of 9241 DVT patients and 453,692 controls, which identified 33 single-nucleotide polymorphisms as instrumental variables of coffee consumption and reported that genetically determined coffee consumption was associated with a slightly elevated DVT risk (odds ratio, 1.01; 95% CI, 1.00-1.02). However, the limited ability of gene variants to capture dietary habits may, to some extent, explain the apparent discrepancy between the results of the Mendelian randomization study and our findings [49].

Coffee and constituents of coffee are known to affect several pathways implicated in the pathogenesis of VTE. In the MEGA study [28], coffee consumers were found to have lower plasma levels of coagulation factor (F)VIII and von Willebrand factor (VWF), both of which are strong and consistent risk factors of VTE [50,51]. Platelets are involved in the pathogenesis of VTE [52,53], and coffee and constituents of coffee (eg, polyphenols) have been shown to inhibit platelet function and platelet aggregation *ex vivo* [[54], [55], [56]]. As the combination of high plasma levels of FVIII and VWF and high platelet reactivity has more than an additive effect on VTE risk [57,58], it might be speculated that the inhibitory effect of coffee on both FVIII/VWF and platelet reactivity would have a synergistic beneficial impact on VTE risk. Further, regular coffee consumption is associated with lower plasma levels of plasminogen activator inhibitor 1, the main inhibitor of fibrinolysis, known to be associated with VTE risk [59,60]. In addition, constituents of coffee, such as caffeine, chlorogenic acid, and polyphenols, have shown anti-inflammatory and antioxidant properties by lowering proinflammatory markers such as C-reactive protein, interleukin-6, and tumor necrosis factor-alpha [61], and neutralizing free radicals and reducing oxidative stress [62], respectively.

Our findings suggest that coffee may potentially lower the risk of VTE. However, due to the nonrandomized observational design of our study, we cannot provide firm recommendations on coffee intake for prevention of VTE at the population level for health policies and guidelines. From another perspective, our findings suggest that coffee intake does not have harmful effects on VTE risk, and that coffee can be safely consumed, potentially with a beneficial effect on VTE.

The main strengths of our study are its prospective design, the large number of participants recruited from 2 cohorts derived from a general population with a high attendance rate, repeated measurements of the exposure and major confounders, a long follow-up time, and validated VTE events. A potential limitation of the study is that several data items, including coffee consumption, were extracted from self-reported questionnaires. Self-reported coffee consumption, however, has been demonstrated to have high validity [45]. Furthermore, although we updated the coffee consumption information at each survey, there could still be fluctuations in an individual’s coffee consumption in the period between surveys that could lead to misclassification and underestimation of the true effect of coffee on VTE risk due to regression dilution bias. The HUNT and Tromsø studies mainly comprised participants of Caucasian descent, and it is therefore unknown whether the results of the study are generalizable to other ethnicities.

In conclusion, we found that coffee consumption was associated with a nonlinear decreased risk of overall VTE and, in particular, PE.
