# Supplementary material for: Repeated measures of coffee consumption and risk of future incident venous thromboembolism—the Trøndelag Health Study and the Tromsø study
Source: Res Pract Thromb Haemost. 2025 Aug 19;9(6):103019. doi: 10.1016/j.rpth.2025.103019 (PMC12570191; doi:10.1016/j.rpth.2025.103019)
Supplement: Supplementary Tables [file mmc1.docx]

**Supplementary Table 1** Incidence rates (IRs) age-adjusted and hazard ratios (HRs) for venous thromboembolism (VTE) in women by categories of daily coffee consumption. The HUNT and Tromsø study 1994-2020

|  | **Person-years** | **VTE** | **Age-adjusted**  **IR (95% CI)** | **Model 1**  **HR (95% CI)** | **Model 2**  **HR (95% CI)** | **Model 3**  **HR (95% CI)** |
| --- | --- | --- | --- | --- | --- | --- |
| **Total VTE** |  |  |  |  |  |  |
| 0 cup per day | 127 107 | 131 | 1.03 (0.86-1.22) | Ref. | Ref. | Ref. |
| 1-2 cups per day | 178 367 | 329 | 1.84 (1.66-2.05) | 0.77 (0.63-0.94) | 0.81 (0.66-1.00) | 0.80 (0.65-0.99) |
| 3-4 cups per day | 330 906 | 617 | 1.86 (1.72-2.02) | 0.74 (0.61-0.90) | 0.77 (0.63-0.94) | 0.75 (0.62-0.91) |
| 5-6 cups per day | 259 557 | 439 | 1.69 (1.54-1.86) | 0.84 (0.69-1.03) | 0.87 (0.71-0.06) | 0.81 (0.66-0.99) |
| > 6 cups per day | 158 976 | 218 | 1.37 (1.20-1.57) | 0.91 (0.73-1.12) | 0.94 (0.76-1.17) | 0.82 (0.65-1.03) |
|  |  |  |  |  |  |  |
| **Provoked VTE** |  |  |  |  |  |  |
| 0 cup per day | 127 107 | 69 | 0.54 (0.43-0.69) | Ref. | Ref. | Ref. |
| 1-2 cups per day | 178 367 | 173 | 0.97 (0.84-1.13) | 0. 77 (0.58-1.02) | 0. 78 (0.59-1.04) | 0.78 (0.58-1.03) |
| 3-4 cups per day | 330 906 | 364 | 1.10 (0.99-1.22) | 0. 83 (0.64-1.08) | 0. 84 (0.64-1.09) | 0.81 (0.62-1.05) |
| 5-6 cups per day | 259 557 | 247 | 0.95 (0.84-1.08) | 0. 90 (0.69-1.18) | 0. 91 (0.69-1.19) | 0.83 (0.63-1.09) |
| > 6 cups per day | 158 976 | 132 | 0.83 (0.70-0.98) | 1.04 (0.78-1.40) | 1.05 (0.79-1.41) | 0.88 (0.65-1.20) |
|  |  |  |  |  |  |  |
| **Unprovoked VTE** |  |  |  |  |  |  |
| 0 cup per day | 127 107 | 62 | 0.49 (0.38-0.63) | Ref. | Ref. | Ref. |
| 1-2 cups per day | 178 367 | 156 | 0.87 (0.75-1.02) | 0.77 (0.57-1.04) | 0.84 (0.62-1.15) | 0.84 (0.62-1.14) |
| 3-4 cups per day | 330 906 | 253 | 0.76 (0.68-0.86) | 0.64 (0.48-0.85) | 0.69 (0.52-0.93) | 0.68 (0.51-0.91) |
| 5-6 cups per day | 259 557 | 192 | 0.74 (0.64-0.85) | 0.78 (0.58-1.04) | 0.83 (0.61-1.11) | 0.79 (0.59-1.07) |
| > 6 cups per day | 158 976 | 86 | 0.54 (0.44-0.67) | 0.75 (0.54-1.04) | 0.81 (0.58-1.14) | 0.74 (0.53-1.05) |

*Age-adjusted incidence rate per 1000 person-years

Model 1: adjusted for age and sex

Model 2: adjusted for age, sex and BMI

Model 3: adjusted for age, sex, BMI, smoking, CVD and Cancer

**Supplementary Table 2** Age-adjusted incidence rates (IRs) and hazard ratios (HRs) for venous thromboembolism (VTE) in men by categories of daily coffee consumption. The HUNT and Tromsø study 1994-2020

|  | **Person-years** | **VTE** | **Age-adjusted**  **IR (95% CI)*** | **Model 1**  **HR (95% CI)** | **Model 2**  **HR (95% CI)** | **Model 3**  **HR (95% CI)** |
| --- | --- | --- | --- | --- | --- | --- |
| **Total VTE** |  |  |  |  |  |  |
| 0 cup per day | 71 177 | 106 | 1.49 (1.23-1.80) | Ref. | Ref. | Ref. |
| 1-2 cups per day | 116 901 | 241 | 2.06 (1.82-2.34) | 0.81 (0.64-1.02) | 0.81 (0.69-0.94) | 0.80 (0.64-1.01) |
| 3-4 cups per day | 244 126 | 524 | 2.15 (1.97-2.34) | 0.83 (0.67-1.03) | 0.81 (0.66-1.01) | 0.81 (0.66-1.00) |
| 5-6 cups per day | 249 409 | 471 | 1.89 (1.73-2.07) | 0.84 (0.68-1.03) | 0.82 (0.67-1.02) | 0.80 (0.65-0.99) |
| > 6 cups per day | 233 914 | 343 | 1.47 (1.32-1.63) | 0.79 (0.64-0.99) | 0.78 (0.63-0.97) | 0.74 (0.59-0.92) |
|  |  |  |  |  |  |  |
| **Provoked VTE** |  |  |  |  |  |  |
| 0 cup per day | 71 177 | 52 | 0.73 (0.56-0.96) | Ref. | Ref. | Ref. |
| 1-2 cups per day | 116 901 | 113 | 0.97 (0.80-1.16) | 0.76 (0.54-1.05) | 0.75 (0.54-1.05) | 0.75 (0.54-1.04) |
| 3-4 cups per day | 244 126 | 294 | 1.20 (1.07-1.35) | 0.93 (0.69-1.25) | 0.91 (0.68-1.22) | 0.90 (0.67-1.22) |
| 5-6 cups per day | 249 409 | 274 | 1.10 (0.98-1.24) | 0. 98 (0.73-1.32) | 0.96 (0.72-1.30) | 0.94 (0.70-1.27) |
| > 6 cups per day | 233 914 | 169 | 0.72 (0.62-0.84) | 0.80 (0.58-1.09) | 0.78 (0.57-1.07) | 0.74 (0.54-1.01) |
|  |  |  |  |  |  |  |
| **Unprovoked VTE** |  |  |  |  |  |  |
| 0 cup per day | 71 177 | 54 | 0.76 (0.58-0.99) | Ref. | Ref. | Ref. |
| 1-2 cups per day | 116 901 | 128 | 1.09 (0.92-1.30) | 0.87 (0.63-1.02) | 0.86 (0.63-1.19) | 0.87 (0.63-1.19) |
| 3-4 cups per day | 244 126 | 230 | 0.94 (0.83-1.07) | 0.73 (0.54-1.00) | 0.72 (0.54-0.97) | 0.72 (0.53-0.97) |
| 5-6 cups per day | 249 409 | 197 | 0.79 (0.69-0.91) | 0.70 (0.52-0.94) | 0.69 (0.51-0.93) | 0.67 (0.50-0.91) |
| > 6 cups per day | 233 914 | 174 | 0.74 (0.64-0.86) | 0.79 (0.58-1.07) | 0.78 (0.57-1.01) | 0.74 (0.54-1.01) |

*Age-adjusted incidence rate per 1000 person-years

Model 1: adjusted for age and sex

Model 2: adjusted for age, sex and BMI

Model 3: adjusted for age, sex, BMI, smoking, CVD and Cancer

**Supplementary Table 3** Baseline characteristics of the HUNT and Tromsø study populations with available information on physical activity (n=158670) across categories of coffee consumption.

| Characteristics | No coffee  consumption | 1-2 cups per day | 3-4 cups per day | 5-6 cups per day | >6 cups per day |
| --- | --- | --- | --- | --- | --- |
| Number of observations | 14 904 | 24 455 | 48 156 | 40 984 | 30 171 |
| Age (years) | 40 ± 16 | 52 ± 18 | 54 ± 15 | 52 ± 14 | 49 ± 13 |
| Women (%, n) | 64 (9 505) | 60 (14 787) | 57 (27 641) | 51 (20 745) | 40 (12 122) |
| BMI (kg m^-2^) | 25.9 ± 4.7 | 26.3 ± 4.3 | 26.5 ± 4.1 | 26.5 ± 4.1 | 26.4 ± 4.1 |
| Smoking (%, n) | 11.6 (1 720) | 9.7 (2 243) | 14.2 (6 847) | 27.1 (11 095) | 45.9 (13 860) |
| Physical activity (%, n) | 76.9 (11 455) | 75.2 (18 394) | 76.2 (36 694) | 75.1 (30 796) | 71.7 (21 634) |
| CVD (%, n) | 3.3 (491) | 8.0 (1 960) | 7.7 (3 706) | 6.6 (2 727) | 5.7 (1 706) |
| Cancer (%, n) | 2.5 (367) | 4.9 (1 209) | 4.9 (2 369) | 4.1 (1 682) | 3.4 (1 010) |
|  |  |  |  |  |  |
| Tromsø |  |  |  |  |  |
| Number of observations | 5 383 | 8 958 | 19 404 | 17 365 | 13 734 |
| Age (years) | 45 ± 15 | 55 ± 16 | 56 ± 14 | 54 ± 13 | 50 ± 13 |
| Women (%, n) | 61 (3 268) | 62 (5 586) | 59 (11 365) | 50 (8 737) | 38 (5 320) |
| BMI (kg m^-2^) | 20.0 ± 4.8 | 26.1 ± 4.4 | 26.2 ± 4.2 | 26.3 ± 4.1 | 26.3 ± 4.2 |
| Smoking (%, n) | 11.9 (640) | 8.2 (734) | 13.0 (2 530) | 24.3 (4 218) | 42.7 (5 868) |
| Physical activity (%, n) | 79.3 (4 267) | 79.1 (7 083) | 79.6 (15 453) | 78.1 (13 560) | 73.0 (10 032) |
| CVD (%, n) | 3.3 (175) | 7.1 (638) | 6.4 (1 243) | 6.1 (1 066) | 5.3 (731) |
| Cancer (%, n) | 3.0 (159) | 4.9 (438) | 4.9 (947) | 4.2 (723) | 3.4 (464) |
|  |  |  |  |  |  |
| HUNT |  |  |  |  |  |
| Number of observations | 9 521 | 15 497 | 28 752 | 23 619 | 16 437 |
| Age (years) | 37 ± 16 | 51 ± 19 | 54 ± 16 | 51 ± 14 | 48 ± 13 |
| Women (%, n) | 66 (6 237) | 59 (9 201) | 57 (16 276) | 51 (12 008) | 41 (6 802) |
| BMI (kg m^-2^) | 25.9 ± 4.7 | 26.3 ± 4.2 | 26.7 ± 4.0 | 26.7 ± 4.0 | 26.6 ± 4.0 |
| Smoking (%, n) | 11.4 (1 089) | 9.7 (1 509) | 15.0 (4 317) | 29.1 (6 877) | 48.6 (7 992) |
| Physical activity (%, n) | 75.5 (7 188) | 73.0 (11 311) | 73.9 (21 241) | 73.0 (17 236) | 70.6 (11 602) |
| CVD (%, n) | 3.3 (316) | 8.5 (1 322) | 8.6 (2 463) | 7.0 (1 661) | 5.9 (975) |
| Cancer (%, n) | 2.2 (208) | 5.0 (771) | 5.0 (1 422) | 4.1 (959) | 3.3 (546) |

**Supplementary Table 4** Age-adjusted incidence rates (IRs) and hazard ratios (HRs) for venous thromboembolism (VTE) by categories of daily coffee consumption among participants who had available information on physical activity (n=158670). The HUNT and Tromsø study 1994-2020

|  | **Person-years** | **VTE** | **Age-adjusted**  **IR (95% CI)*** | **Model 1**  **HR (95% CI)** | **Model 2**  **HR (95% CI)** | **Model 3**  **HR (95% CI)** | **Model 4**  **HR (95% CI)** |
| --- | --- | --- | --- | --- | --- | --- | --- |
| **Total VTE** |  |  |  |  |  |  |  |
| 0 cup per day | 180675 | 212 | 2.40 (2.06-2.72) | Ref. | Ref. | Ref. | Ref. |
| 1-2 cups per day | 263030 | 457 | 1.76 (1.60-1.92) | 0.72 (0. 61-0.86) | 0.74 (0.62-0.87) | 0.74 (0.62-0.87) | 0.73 (0.62-0.87) |
| 3-4 cups per day | 510476 | 932 | 1.77 (1.66-1.89) | 0.73 (0. 63-0.85) | 0.73 (0.63-0. 85) | 0.73 (0.63-0. 85) | 0.72 (0.62-0.84) |
| 5-6 cups per day | 453977 | 746 | 1.90 (1.76-2.04) | 0.77 (0. 66-0.90) | 0.77 (0.66-0.90) | 0.77 (0.66-0.90) | 0.73 (0.63-0.86) |
| > 6 cups per day | 352929 | 499 | 2.03 (1.85-2.22) | 0.81 (0.69-0.95) | 0.82 (0.69-0.96) | 0.81 (0.69-0.96) | 0.74 (0.63-0.87) |
| **Provoked VTE** |  |  |  |  |  |  |  |
| 0 cup per day | 180675 | 105 | 1.19 (0.96-1.43) | Ref. | Ref. | Ref. | Ref. |
| 1-2 cups per day | 263030 | 222 | 0.86 (0.74-0.97) | 0.70 (0.55-0.89) | 0.70 (0.55-0.88) | 0.70 (0.55-0.88) | 0.69 (0.55-0.87) |
| 3-4 cups per day | 510476 | 543 | 1.03 (0.95-1.12) | 0.85 (0.68-1.05) | 0.83 (0.67-1.03) | 0.84 (0.68-1.03) | 0.82 (0.66-1.01) |
| 5-6 cups per day | 453977 | 421 | 1.08 (0.97-1.18) | 0.88 (0.71-1.09) | 0.86 (0.69-1.07) | 0.86 (0.69-1.07) | 0.81 (0.65-1.00) |
| > 6 cups per day | 352929 | 266 | 1.09 (0.96-1.22) | 0.89 (0.71-1.11) | 0.88 (0.70-1.10) | 0.87 (0.70-1.09) | 0.77 (0.61-0.97) |
| **Unprovoked VTE** | |  |  |  |  |  |  |
| 0 cup per day | 180675 | 107 | 1.20 (0.97-1.43) | Ref. | Ref. | Ref. | Ref. |
| 1-2 cups per day | 263030 | 235 | 0.91 (0.79-1.02) | 0.75 (0.60-0.95) | 0.78 (0.62-0.99) | 0.78 (0.62-0.99) | 0.78 (0.62-0.99) |
| 3-4 cups per day | 510476 | 389 | 0.74 (0.67-0.81) | 0.61 (0.49-0.76) | 0.63 (0.50-0.78) | 0.63 (0.50-0.78) | 0.62 (0.50-0.77) |
| 5-6 cups per day | 453977 | 325 | 0.83 (0.74-0.92) | 0.67 (0.54-0.83) | 0.68 (0.54-0.85) | 0.68 (0.54-0.85) | 0.66 (0.53-0.82) |
| > 6 cups per day | 352929 | 233 | 0.94 (0.82-1.07) | 0.74 (0.59-0.93) | 0.75 (0.60-0.95) | 0.75 (0.60-0.96) | 0.71 (0.56-0.90) |
| **DVT** |  |  |  |  |  |  |  |
| 0 cup per day | 180675 | 103 | 1.13 (0.92-1.35) | Ref. | Ref. | Ref. | Ref. |
| 1-2 cups per day | 263030 | 242 | 0.94 (0.82-1.05) | 0.83 (0. 65-1.04) | 0.84 (0.67-1.07) | 0.84 (0.67-1.07) | 0.84 (0.66-1.06) |
| 3-4 cups per day | 510476 | 496 | 0.95 (0.86-1.03) | 0.83 (0.67-1.03) | 0.84 (0.67-1.04) | 0.84 (0.67-1.04) | 0.82 (0.66-1.02) |
| 5-6 cups per day | 453977 | 396 | 1.00 (0.90-1.00) | 0.86 (0.69-1.08) | 0.87 (0.69-1.08) | 0.87 (0.70-1.08) | 0.82 (0.66-1.02) |
| > 6 cups per day | 352929 | 286 | 1.14 (1.00-1-28) | 0.96 (0.77-1.21) | 0.97 (0.78-1.22) | 0.97 (0.77-1.22) | 0.88 (0.69-1.11) |
| **PE** |  |  |  |  |  |  |  |
| 0 cup per day | 180675 | 109 | 1.27 (1.02-1.51) | Ref. | Ref. | Ref. | Ref. |
| 1-2 cups per day | 263030 | 215 | 0.83 (0.72-0.94) | 0.63 (0.50-0.80) | 0.64 (0.50-0.80) | 0.63 (0.50-0.80) | 0.63 (0.50-0.80) |
| 3-4 cups per day | 510476 | 436 | 0.83 (0.75-0.91) | 0.63 (0.51-0.78) | 0.64 (0.51-0.79) | 0.64 (0.51-0.79) | 0.63 (0.51-0.78) |
| 5-6 cups per day | 453977 | 350 | 0.90 (0.81-1.00) | 0.68 (0.55-0.85) | 0.68 (0.55-0.84) | 0.68 (0.55-0.84) | 0.65 (0.52-0.81) |
| > 6 cups per day | 352929 | 213 | 0.89 (0.77-1.01) | 0.67 (0.53-0.85) | 0.67 (0.53-0.85) | 0.67 (0.53-0.84) | 0.61 (0.48-0.78) |

*Age-adjusted incidence rate per 1000 person-years

Model 1: adjusted for age and sex

Model 2: adjusted for age, sex and BMI

Model 3: adjusted for age, sex, BMI and physical activity

Model 4: adjusted for age, sex, BMI, smoking, physical activity, CVD and Cancer
